# Difficult Cases

**Published:** 1888-02-11

**Authors:** J. Wilson

**Affiliations:** Hon. Sec. Workhouse Nursing Association. 45, Colville Gardens, W.


### DIFFICULT CASES.

I AM anxious to know the addresses of homes which would be likely to help in the following case: An illegitimate child —a girl—is now in a country workhouse. The mother is a person of very bad character, who, if she removes the child, will be certain to bring it up as ill as it is possible to conceive. The age of the child is only four—too young for the "waif and stray" homes. No payment can be made. Are there any homes which could help under the circumstances?

J. WILSON,
Hon. Sec. Workhouse Nursing Association.
45, Colville Gardens, W.

[We shall be glad to hear of a suitable place for this worse than orphaned child.—ED. *T. H.*]

